# Functional network mediates age-related differences in reaction time: a replication and extension study

**DOI:** 10.1002/brb3.324

**Published:** 2015-03-13

**Authors:** Yunglin Gazes, Christian Habeck, Deirdre O'Shea, Qolamreza R Razlighi, Jason Steffener, Yaakov Stern

**Affiliations:** Cognitive Neuroscience Division, Department of Neurology, Columbia University College of Physicians and SurgeonsP&S Box 16, 630 West 168th Street, New York, New York, 10032

**Keywords:** Aging, functional magnetic resonance imaging, mediation, ordinal trend covariance analysis, study replication, task-switching

## Abstract

**Introduction:**

A functional activation (i.e., ordinal trend) pattern was previously identified in both young and older adults during task-switching performance, the expression of which correlated with reaction time. The current study aimed to (1) replicate this functional activation pattern in a new group of fMRI activation data, and (2) extend the previous study by specifically examining whether the effect of aging on reaction time can be explained by differences in the activation of the functional activation pattern.

**Method:**

A total of 47 young and 50 older participants were included in the extension analysis. Participants performed task-switching as the activation task and were cued by the color of the stimulus for the task to be performed in each block. To test for replication, two approaches were implemented. The first approach tested the replicability of the predictive power of the previously identified functional activation pattern by forward applying the pattern to the Study II data and the second approach was rederivation of the activation pattern in the Study II data.

**Results:**

Both approaches showed successful replication in the new data set. Using mediation analysis, expression of the pattern from the first approach was found to partially mediate age-related effects on reaction time such that older age was associated with greater activation of the brain pattern and longer reaction time, suggesting that brain activation efficiency (defined as “the rate of activation increase with increasing task difficulty” in *Neuropsychologia* 47, 2009, 2015) of the regions in the Ordinal trend pattern directly accounts for age-related differences in task performance.

**Discussion:**

The successful replication of the functional activation pattern demonstrates the versatility of the Ordinal Trend Canonical Variates Analysis, and the ability to summarize each participant's brain activation map into one number provides a useful metric in multimodal analysis as well as cross-study comparisons.

## Introduction

Task-switching has been frequently studied in the context of aging due to its sensitivity to differences in executive functions. An important step toward fully understanding the effect of aging on executive functions is to explore the neural factors that contribute to behavioral change. One such neural factor that is closely tied to behavioral performance is the activation of a set of functionally connected but spatially distributed regions across the brain, hypothesized to show temporally correlated activity throughout task performance. Relating differential expression of this functional activation pattern to differences in task performance across young and older adults can provide insight into a neural system that is vulnerable to age-related decline. In Gazes et al. (2012), we described a functional connectivity pattern in functional Magnetic Resonance Imaging (fMRI) data that was associated with task-switching and whose expression was related to behavioral performance. The current study aimed to (1) replicate this pattern in a new group of activation data, and (2) extend the previous study by directly examining whether the effect of aging on task performance can be explained, or mediated, by the activation of this functional pattern.

The previously reported functional activation pattern was identified using Ordinal Trend Canonical Variates Analysis (OrT CVA) (Habeck et al. 2004, 2005a,b), which is a multivariate technique that aims at deriving group-invariant spatial covariance patterns, similar to other analysis techniques based on Principal Components Analysis. OrT CVA applies a stringent search criterion to find a set of correlated brain regions, the pattern expression of which changes monotonically (consistently) with increasing task load. Monotonicity requirement in pattern expression is based on the assumption that any task-related brain activity should change with greater task load. The functional pattern extracted by OrT CVA consists of brain regions with mutually correlated brain activity while performing task-switching. The degree of pattern expression is quantified by an expression score and can be used to examine its relationship with task performance.

Using OrT CVA, we identified a task-switching pattern that consisted of both regions with positive and with negative loadings. Activation in the regions with positive loadings increases with transition from the single-task to the task-switching condition, while regions with negative loadings decrease in activity with increasing demand. Table 1 shows the regions with a *z*-value >1.96 and an extent threshold (*k*) of 50 voxels in the OrT pattern identified in Gazes et al. (2012), which includes regions with both positive and negative weights. Due to the block-based design in the fMRI modeling, which maximizes the chance of replicability by optimizing the signal-to-noise ratio, the pattern consisted of regions involved in all aspects of task-switching performance, from basic visuomotor tasks to higher cognitive control processes. As a validation of the relationship of the OrT pattern to important aspects of task performance, change in expression of this pattern from single- task to task-switch conditions correlated with the difference between single-task and task-switch conditions or ΔRT.

**Table 1:** Brain regions exceeding the threshold of *z* > 1.96 and extent threshold of 50 contiguous voxels in the Study I Ordinal Trend pattern

Region	Lat	BA	*x*	*y*	*z*	*k*
Positive Loading						
Prefrontal cortex	L	9	−38	20	40	129
Mid-occipital/Precuneus	L	7/19	−30	−78	36	251
Cerebellum	L/R	NA	−20	−74	−26	4301
Thalamus	R	NA	2	−14	12	51
Superior parietal lobule	R	7	26	−74	46	114
Occipital gyrus	R	19	34	−78	28	133
Negative Loading						
Middle temporal gyrus	L	37	−42	−64	0	53
Superior temporal gyrus/Claustrum	L	38	−36	−6	−10	367
Putamen	L	NA	−24	−4	10	79
Posterior cingulate	L	26	−8	−44	28	66
Medial frontal gyrus	R	10	0	50	6	56
Cingulate gyrus	L/R	24	4	−8	42	61
Supplementary Motor Area	R	6	6	−18	54	63
Anterior Cingulate	R	24	6	28	24	56
Superior temporal gyrus/hippocampus	R	38	36	2	−16	149
Precentral gyrus/Insula cortex	R	6/13	48	−8	10	542
Inferior frontal gyrus	R	45/47	50	34	2	77
Postcentral gyrus/Sup temporal gyrus	R	40/42	62	−24	20	140

Lat, laterality; BA, Brodmann Area; L, left; R, right; NA, not applicable; *x*,*y*,*z*, MNI coordinates; *k*, cluster size.

Replication of results is an essential part of research that is too frequently ignored. This is particularly essential in fMRI research, given the high level of noise in fMRI data (Ramsey et al. 2002) as well as the inflation of alpha level due to multiple comparisons across thousands of voxels in activation images. An advantage of the OrT CVA is that OrT patterns can be readily forward applied to new activation data in order to evaluate the reproducibility of the result. Expression scores of the new activation data for the OrT pattern can then be related to performance and also tested for age effects. Successful replication of the brain pattern strengthens the validity of the functional connectivity pattern. Once validated, the pattern can be forward applied to other task-switching fMRI data as a standard measure for cross-study comparison. However, whereas this replication method is actually replication of the predictive ability of the original OrT pattern, an alternative replication method would be to derive a pattern from the new data set and then determine the statistical uniqueness of the spatial correlation between the original and the new pattern. This was done by comparing the resulting spatial correlation to a distribution of the spatial correlation values obtained from randomly permuting group membership between the two studies. The spatial correlation between the two studies falling above the left tail of the permuted spatial correlation histogram would be evidence that the two OrT patterns show the same degree of similarity as patterns derived from intermixing the participants from Studies I and II, and thus are not statistically dissimilar from each other.

In addition to replication, the current study used mediation analysis to evaluate whether age-related differences in behavior can be attributed to differences in the degree of expression for the fMRI pattern. This is a more stringent and specific test of the relationship among age, fMRI activation, and task performance. Aging has been shown to negatively affect task performance (Salthouse 2009), and older adults have been found to show greater activation than younger adults and also to sometimes activate additional brain regions (Grady 2008). However, only a few studies have directly explored whether age-related differences in performance can be explained by differences in brain activation. Examination of the relationship between age-related differences in performance and in brain activation can be performed with mediation analysis. Following Baron and Kenny's (1986) mediation analysis on task-switching data, Madden 2010 demonstrated that functional connectivity during cue processing mediated age-related decline in drift rate, which is a measure of the efficiency of target categorization. Older adults showed weaker connectivity and poorer cognitive efficiency. Rather than examining functional connectivity, Steffener et al. (2014) used a novel voxel-wise mediation analysis which located brain regions where activation mediated the effect of age on task-switching performance. This approach identified the set of regions whose age-related neural activity partially explained the variance in the age-related differences in task performance.

The current study also used mediation to examine whether neural activation accounts for age-related differences in task performance, but rather than on a voxel-wise basis, the expression of the OrT pattern was used for the fMRI activation measure. By relating age and task performance to the expression of the OrT pattern, we tested the hypothesis that activation of a set of previously identified functionally connected regions explains the age-related differences in task performance. Our approach does not generate a new set of brain regions that shows a mediation effect. Rather, the brain regions were already selected prior to the mediation and only the mediational effect of the pattern expression for age and task performance was tested.

In summary, the current paper presented the validation of the OrT pattern in a new data set and demonstrated that load-related changes in the expression of this fMRI pattern mediates the effect of age on load-related changes in reaction time. Relating the expression of a replicated fMRI pattern to a behavioral measure further validated the reliability of the pattern and can significantly enhance our understanding of neural changes responsible for this important feature of cognitive aging.

## Method

### Participants

Forty three participants from the original study (here called Study I) and 54 new participants (here called Study II) were included in the current analyses for a total of 47 younger and 50 older participants. Table 2 shows details of the participants' demographic information. Participants were recruited using established market mailing procedures to equalize the recruitment procedures of young and old adults. Participants who responded to the mailing were telephone screened to ensure that they met basic inclusion criteria (right handed, English speaking, no psychiatric or neurological disorders, normal or corrected-to-normal vision, etc.). Individuals that passed the telephone screen were further screened in person and a Mattis Dementia Rating Scale score of at least 133 was required for retention in the study. Informed consent, as approved by the Internal Review Board of the College of Physicians and Surgeons of Columbia University, was obtained prior to study participation, and after the nature and risks of the study were explained. Participants were paid for their participation in the study.

**Table 2:** Participant information

	Study I		Study II	
	Young	Old	Young	Old
*N*	22	21	25	29
Age (years)^1^	25 ± 2.8	65 ± 2.5	27 ± 2.7	65 ± 2.8
Gender	10 M, 12 F	9 M, 12 F	9 M, 16 F	11 M, 18 F
Education (years)^1^	15 ± 2.2	15 ± 3.2	15 ± 1.9	16 ± 2.9
DRS^1^	141 ± 2.4	141 ± 2.8	140 ± 2.3	140 ± 2.6

1 Mean ± Standard error.

M, males; F, females; DRS, Mattis Dementia Rating Scale.

### Activation task

**Figure 1:**
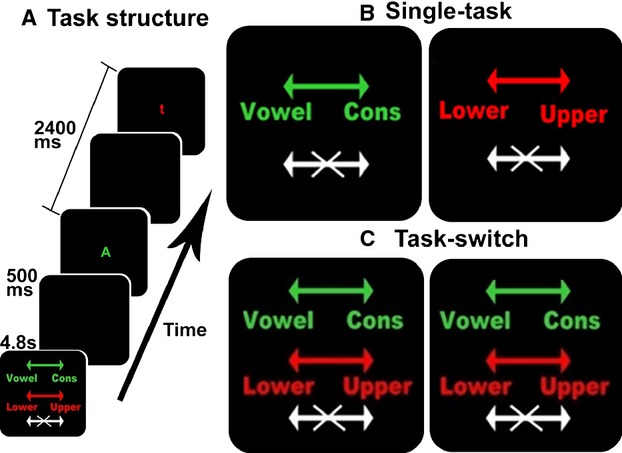
Illustration of the activation task. (A) Temporal structure of the task. (B) Instruction for the single-task condition. (C). Instruction for the task-switch condition. The arrows indicate left versus right-hand response. Vowel versus consonant indicate discrimination of vowel/consonant and lower versus upper indicate discrimination of lower/upper-case.

The task performed during fMRI acquisition was based on Experiment 2 described by Koechlin et al. (2003), which is an intrinsically cued task-switching paradigm with a no-go component (for examining a motoric factor not reported in this study). The intrinsic cue was the color of the stimulus, denoting one of three possible tasks: upper/lower-case discrimination (red), vowel/consonant discrimination (green), and no-go trial (white). In the single-task condition, the two discrimination tasks were administered as separate blocks, with eight trials in each block. In the task-switching condition, on average, four switch and four repeat trials were randomly distributed across each of the two blocks. Six runs were performed in the fMRI with two blocks of each condition per run (i.e., two blocks each of the two discrimination tasks alone, and two task-switch blocks). Each stimulus was terminated when a response was made before deadline or after 1900 ms, whichever came first. Stimuli onsets were separated by 2400 ms. Together, each block was 33.6 sec long preceded by a 4.8 sec instruction cue informing subjects of the appropriate action for each stimulus. Subjects responded to each letter with a right-hand/left-hand button press or by making no action at all. Each participant also completed six identical runs before entering the scanner. See Figure1 for details of the task.

### MRI acquisition

MRI images were acquired in a 3.0T Philips Achieva Magnet using a standard quadrature head coil. A T1-weighted scout image was acquired to determine subject position. One hundred and sixty-five contiguous 1 mm coronal T1-weighted images of the whole brain were acquired for each subject with an MPRAGE sequence using the following parameters: TR 6.5 ms, TE 3 ms; flip angle 8°, acquisition matrix 256 × 256 and 240 mm field of view. Six functional scan sets were acquired, each of which included collection of 111 functional images acquired using a field echo-echo planar imaging (FE-EPI) sequence TE/TR = 20 ms/2000 ms; flip angle = 72°; 112 × 112 matrix; in-plane voxel size = 2.0 mm × 2.0 mm; slice thickness = 3.0 mm (no gap); 41 transverse slices per volume. Before the initiation of the activation task, four volumes were acquired and discarded to allow transverse magnetization immediately after radio-frequency excitation to approach its steady-state value.

Any T1 scans with potentially clinically significant findings, such as abnormal neural structure, were reviewed by a neuroradiologist and removed from the sample prior to the current analysis. However, no clinically significant findings were identified or removed.

### fMRI analysis

All image preprocessing and statistical analyses for fMRI data were implemented using the SPM8 program (Wellcome Department of Cognitive Neurology) and other in-house code written in MatLab 7.10 (Mathworks, Natick MA). Images were visually inspected for imaging artifacts and neurological pathology and manually aligned along the AC-PC line. For each of the six functional data sets (corresponding to the six block types in the experimental design) from each participant, images were temporally shifted to correct for slice acquisition order using the middle slice acquired in the TR as the reference and were corrected for motion by realigning to the first volume of the first session. The T1-weighted (structural) image was coregistered to the first functional volume using mutual information co-registration algorithm implemented in SPM8. This coregistered high-resolution image was then used to determine the 7 × 8 × 7 nonlinear basis function parameters for transformation into a standard space defined by the Montreal Neurologic Institute (MNI) template brain supplied with SPM8. This transformation was then applied to the functional data, which were resliced using sinc-interpolation to 2 × 2 × 2 mm and finally spatially smoothed with an 8 mm FWHM kernel. Both the data and the time-series design matrix were high-pass filtered with a filter cutoff period of 128 sec. Autocorrelations within the time-series were corrected for by prewhitening the data.

For the first level analysis, a block-based analysis was performed on each participant's fMRI data in which the predictor variables in the design matrix were composed of epochs representing each unique experimental task block; each of six runs was separately modeled within which one predictor for each of four task blocks and one predictor for instructions were modeled. (The rest blocks were implicitly modeled) Each epoch was convolved with a model of the hemodynamic response function supplied with SPM8. Contrast images, which are linear combinations of the corresponding beta maps across the six blocks, for single-task and task-switch conditions were entered into the group-level analysis.

Group-level analysis was performed on data from Study II by using a forward application of the task-switching pattern derived in Study I as reported in Gazes et al. (2012). This task-switching pattern was derived using a software created in Matlab called Generalized Covariance Analysis (gCVA: http://www.nitrc.org/projects/gcva_pca) (Habeck et al. 2005a). In our previous report, an OrT pattern was identified in both age groups whose expression increased monotonically from the single-task to the task-switching condition. The assumption that underlies this analysis is that activation in task-related regions tend to vary with task difficulty, thus by extracting regions whose activation changes from the single-task to the task-switch condition, a set of regions that are involved in task processing are extracted.

### Statistical analysis

#### Test for replication of the predictive ability of the Study I OrT pattern

To test for replication of this task-related fMRI pattern identified in Study I, the pattern was mathematically projected onto independent data acquired for the same task in Study II participants by calculating the dot product of each individual's single-task and task-switch contrast maps (linear combination of beta maps), separately, with the point estimate of Study I's OrT pattern. This produced expression scores for task-switching pattern in each of the new subjects for both task conditions. To ascertain whether the task-switching pattern exhibits a statistically significant ordinal trend (monotonic change in the expression scores from single-task to task-switch condition) (Habeck et al. 2005a) in the independent data, we conducted a permutation test in which the task data were resampled and the condition assignment (single-task/task-switch) was broken, while leaving the subject assignment intact. The test statistic was the number of exceptions to the ordinal trend, that is, the number of subjects who failed to show a monotonic increase in their level of expression of the task-switching pattern from the single-task to the task-switch condition. This was computed by executing the permutation 10,000 times to generate a null-hypothesis histogram for the ordinal-trend statistic. The *P*-level is then read off as the fraction of the iterations that produced a statistic smaller than, or equal to, our point estimate value. This shows whether the number of exceptions to the ordinal trend is smaller than that expected by chance. A *P*-threshold of 0.05 was chosen for testing statistical significance.

#### Derivation of a new OrT pattern in Study II data

A new OrT pattern was identified in Study II data independently of the original OrT pattern to test the spatial replicability. The unique aspect of OrT CVA is that it aims to derive covariance patterns that show increasing activation with task demand in parametric task designs on a subject-by-subject basis, rather than just showing mean task-related increases that allow for substantial numbers of subjects to deviate from the required monotonic change across task demand levels. The within-subject monotonic change in pattern expression is a powerful constraint that minimizes type-I error, and the number of subjects who violate the monotonic pattern expression demand (number of exceptions) are used as an inferential test statistic for computing a null-histogram from permuted data in which the subject assignment is intact, but the task assignment has been randomized. An OrT pattern is significant if the number of exceptions to the monotonicity constraint is less than that expected by chance (calculated by [the number of exceptions in permuted data less than the point estimate]/[total number of permutations]). In addition to ascertaining whether a significant ordinal trend is present, there is also the question of the robustness of loadings in the ordinal-trend pattern; this is assessed with a bootstrap resampling procedure, in the form of a *Z*-statistics map with both positive and negative values.

True replication of the OrT pattern requires the two independently derived patterns to show good spatial similarity. To test for spatial similarity, a permutation test was performed in which participants from each study were permuted across the two studies. For each permutation, two OrT patterns were derived and the spatial correlation calculated. Spatial correlation of the patterns from the unpermuted data sets was compared with the spatial correlations of the permuted data. Replication of the OrT pattern was considered successful if the spatial correlation of the unpermuted data lies within the body of the permuted correlation histogram not in the left tail, indicating that the unpermuted data samples give rise to patterns that are not significantly less similar than the permuted samples.

#### Behavioral analyses for Study II

To observe the main effects of Age group and Task condition on task performance and fMRI expression in Study II data, two task performance scores, reaction time (RT) and accuracy, and fMRI pattern expression scores were tested separately in three models for Age Group, Task Condition (single-task/task-switch) and their interaction effects using Repeated-measures Analysis of Variance (ANOVA). In another set of analyses, the difference between the two task conditions was calculated for RT (ΔRT), accuracy (ΔAccuracy), and fMRI expression (ΔfMRI expression) and the effect of Age group was examined. All statistical analyses were performed in IBM SPSS v. 19.

#### Expression scores predicting task performance

To examine whether pattern expression scores predict task performance (accuracy and RT) after accounting for Age group and Condition effects, the method of heterogeneous slopes (Siegel 1956; Kumar et al. 2008) was used. The analysis started with a general linear model that included all of the possible interaction terms in addition to the main effects and then the model was reduced by excluding all nonsignificant interactions. RT and accuracy were the dependent variables in two separate models. Each full model was comprised of seven predictors: Age group, Condition, Pattern expression, Age group × Condition, Age group × Pattern expression, Condition × Pattern expression, and Age group × Condition × Pattern expression. Age differences in the relationship between Pattern expression and task performance would be supported by a significant interaction involving Age group and Pattern expression.

Variables measuring the change between the two task conditions were tested in analogous models: ΔfMRI expression and Age group predicting ΔRT and ΔAccuracy in separate models.

#### Mediating effect of fMRI expression score

As a follow-up analysis to the difference of slopes test, for any task performance measure that was significantly predicted by pattern expression, mediation analysis was performed. If the relationship was similar between the two studies, data from both studies were combined in the mediation analysis after study effects were tested to be nonsignificant. The model consisted of Age group as the independent variable and task performance as the dependent variable, and fMRI pattern expression score was the mediating variable. Age group was dummy-coded with younger adults equaling to 1s and older adults were 2s. The PROCESS macro for SPSS (Hayes 2013) was used. 5000 stratified bootstrap resamples were made to determine the bias-corrected percentile confidence intervals. Confidence intervals excluding 0 were evidence of significant mediation effect.

## Results

### Behavioral analysis for Study II

To test for experimental main effects on task performance, accuracy and RT were separately modeled with Age group and Condition as independent variables. Performance on task-switching showed the expected trends for Condition and Age Group. Proportion of accurate trials decreased from 0.962 (95% CI [0.951, 0.972]) in single-task to 0.909 (95% CI [0.890, 0.928]) in task-switch condition and also was higher in younger participants, 0.954 (95% CI [0.934, 0.974]), than in older participants, 0.917 (95% CI [0.898, 0.935]). Table 3 shows the *F* and *t* statistics for all main effects and interactions. Interaction of Condition by Age group was significant such that younger participants' accuracy decreased from 0.968(95% CI [0.953, 0.983]) to 0.939(95% CI [0.912, 0.967]) while older participants' accuracy decreased from 0.955(95% CI [0.941, 0.969]) to 0.879(95% CI [0.853, 0.904]). Similarly, reaction time (RT) increased from 768 (95% CI [741, 795]) ms in single-task to 1046 (95% CI [997, 1095]) ms in task-switch condition and also was shorter in younger participants, 806 (95%CI [753, 859]) ms, than in older participants, 1007 (95% CI [958, 1056]) ms. Significant Condition by Age gsroup Interaction was observed where younger participants' RT slowed from 691 (95% CI [651, 731]) ms in single-task to 921 (95% CI [850, 993]) ms in the task-switch condition while older participants RT slowed from 844 (95% CI [807, 881]) ms in single-task to 1170 (95% CI [1104, 1237]) ms in task-switch condition.

The accuracy and RT differences between single-task and task-switch conditions, delta accuracy, and ΔRT, respectively, were tested for Age group effects, and both measures showed significant Age group effect (ΔAccuracy: 95% CI of Difference [0.0229, 0.0784]; ΔRT: 95% CI of Difference [31.1, 160]). Across the two conditions, older participants showed greater ΔAccuracy (M = 0.077; 95% CI [0.055, 0.098]), than younger participants (M = 0.026; 95% CI [0.0087, 0.043]), and ΔRT was also greater in older (M = 326 ms; 95% CI [273, 379]) than in younger participants (M = 230 ms; 95% CI [196, 265]).

### Replication of fMRI pattern

Forward application of the fMRI pattern observed in Study I to Study II showed successful replication of the pattern derived from Study I. Success was determined by whether the expression scores in Study II follow a statistically significant ordinal trend such that the majority of the participants showed a monotonic increase in the expression of the pattern from the single-task to the task-switch condition. Out of 54 participants, the number of exceptions to the ordinal trend was 11. This corresponded to *P *<* *0.0001 in the bootstrap test, indicating that the number of exceptions was significantly smaller than that expected by chance. In a repeated-measures ANOVA in which the expression scores were dependent variables, and Condition and Age group were independent variables, Condition effect was significant, such that expression scores increased from single-task (M = 0.274, 95% CI [0.217, 0.332) to task-switch condition (M = 0.387, 95% CI [0.319, 0.456]). Age group also had an effect on expression scores in which younger participants had mean scores of 0.211 (95% CI [0.123, 0.300]) and older participants had a mean score of 0.387 (95% CI [0.319, 0.456]). Age group by Condition interaction was not significant. The expression difference from the two task conditions (ΔfMRI expression) also did not show an Age group effect. See Table 3 for the *F* and *t* statistical values.

**Table 3:** *F* and *t* statistics for all main effects and interactions

Dependent Variable	Age Group	Condition	Age × Cond	fMRI	Cond × fMRI	Age × fMRI	Age × Cond × fMRI
Experimental main effects on task performance							
Accuracy	7.45**	60.5***	12.1**	na	na	na	na
RT	31.2***	301.9***	8.88**	na	na	na	na
ΔAccuracy	3.66**	na	na	na	na	na	na
ΔRT	2.98**	na	na	na	na	na	na
fMRI	15.9***	10.6**	1.52	na	na	na	na
ΔfMRI	1.23	na	na	na	na	na	na
fMRI predicting task performance							
Accuracy	13.0***	27.1***	2.23	1.66	0.007	0.026	0.058
RT	39.6***	94.1***	2.14	<0.001	0.583	0.09	0.743
ΔAccuracy	11.8**	na	na	1.28	na	0.888	na
ΔRT	7.15*	na	na	4.69*	na	0.757	na

*P <* *0.05; **P <* *0.01; ***P <* *0.001; na, not applicable.

The 11 participants that exhibited exceptions to the Ordinal Trend (i.e., pattern expression did not increase from single-task to Task-switch condition) consisted of seven older and four younger adults but their accuracy and RT were not significantly different from nonexception participants (*P* > 0.05 for single, task-switch, and Δaccuracy and ΔRT). However, as shown in section 3.4, excluding these participants from mediation analysis improved the overall mediation effect.

### Expression scores predicting task performance

Replication of Study I results was also observed in the relationships between fMRI expression and behavior evaluated by the difference of slopes tests. For RT and Accuracy, the independent variables were Age group, Condition, and fMRI expression scores. The fMRI expression scores in each of the two task conditions did not predict the respective Accuracy or RT. For ΔRT and ΔAccuracy (change in performance from single-task to task-switch blocks), independent variables were Age group and ΔfMRI expression scores. Similar to results reported in Gazes et al. (2012), ΔfMRI expression scores of the OrT pattern predicted ΔRT even after accounting for Age group effects, but did not predict ΔAccuracy. See Table 3 for F statistics.

### Mediating effect of fMRI expression score

**Figure 2:**
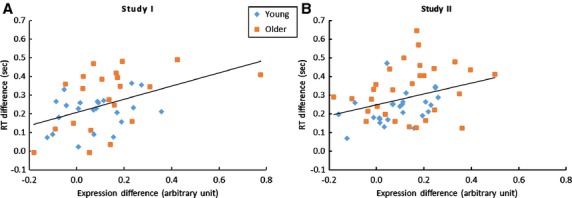
Scatter plot of ΔRT versus ΔfMRI expression in Study 1 (A) and Study 2 (B).

**Figure 3:**
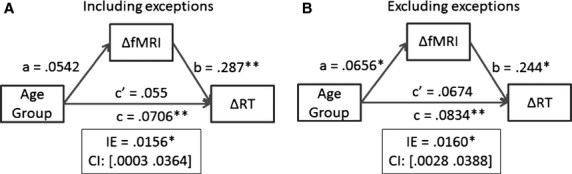
Illustration of the mediation of Age group on ΔRT by ΔfMRI expression. Model including OrT exception participants is shown in A while B shows the model excluding the exceptions. a = beta for the effect of Age group on ΔfMRI expression; b = beta for the effect of ΔfMRI on ΔRT; c = total effect of Age group on ΔRT; c'=effect of Age group on ΔRT controlling for ΔfMRI; IE = indirect effect of Age group on ΔRT through ΔfMRI; CI = 95% confidence interval for the indirect effect. *p < 0.05; **p < 0.01.

As a follow-up to the difference of slopes test which showed evidence that ΔfMRI expression scores predicted ΔRT, mediation analysis was performed to examine whether age-related differences in ΔRT can be explained by differences in ΔfMRI expression scores. Given that similar relationship between ΔfMRI and ΔRT was observed in both Study I and II data (see Fig.2), and there were no study differences on ΔfMRI expression scores (*t*_95_ = 0.262, *n.s*.) or on ΔRT (*t*_95 _= 1.38, *n.s*.), data from Study I and II were combined in the mediation analysis. The analysis indicated that ΔfMRI expression scores mediated the effect of Age group on ΔRT. As shown in Figure3A, older participants had higher ΔfMRI expression scores and greater ΔRT (coefficient of *a*) but this effect was marginally significant (*P *=* *0.0814), and higher ΔfMRI expression scores were associated with greater ΔRT (coefficient of *b*, *P *=* *0.0004). Direct effect of Age group on ΔRT (coefficient of c*'*) was also significant (*P *=* *0.0041). In order to test whether the mediational relationship improves if the analysis was restricted to only the participants who showed an ordinal trend in their brain activation, mediation of age effect on ΔRT through ΔfMRI expression scores was reanalyzed by excluding participants who were exceptions to the OrT. Not only was the mediation effect stronger, shown in Figure3B, the relationship between Age group and ΔfMRI also gained statistical significance (*P* = 0.0257). Overall the significant indirect effect of Age group on ΔRT via ΔfMRI expression scores suggests that Age group exerts some of its influences on task performance through differences in ΔfMRI expression scores.

### Study II OrT pattern and its spatial similarity to Study I Pattern

**Figure 4:**
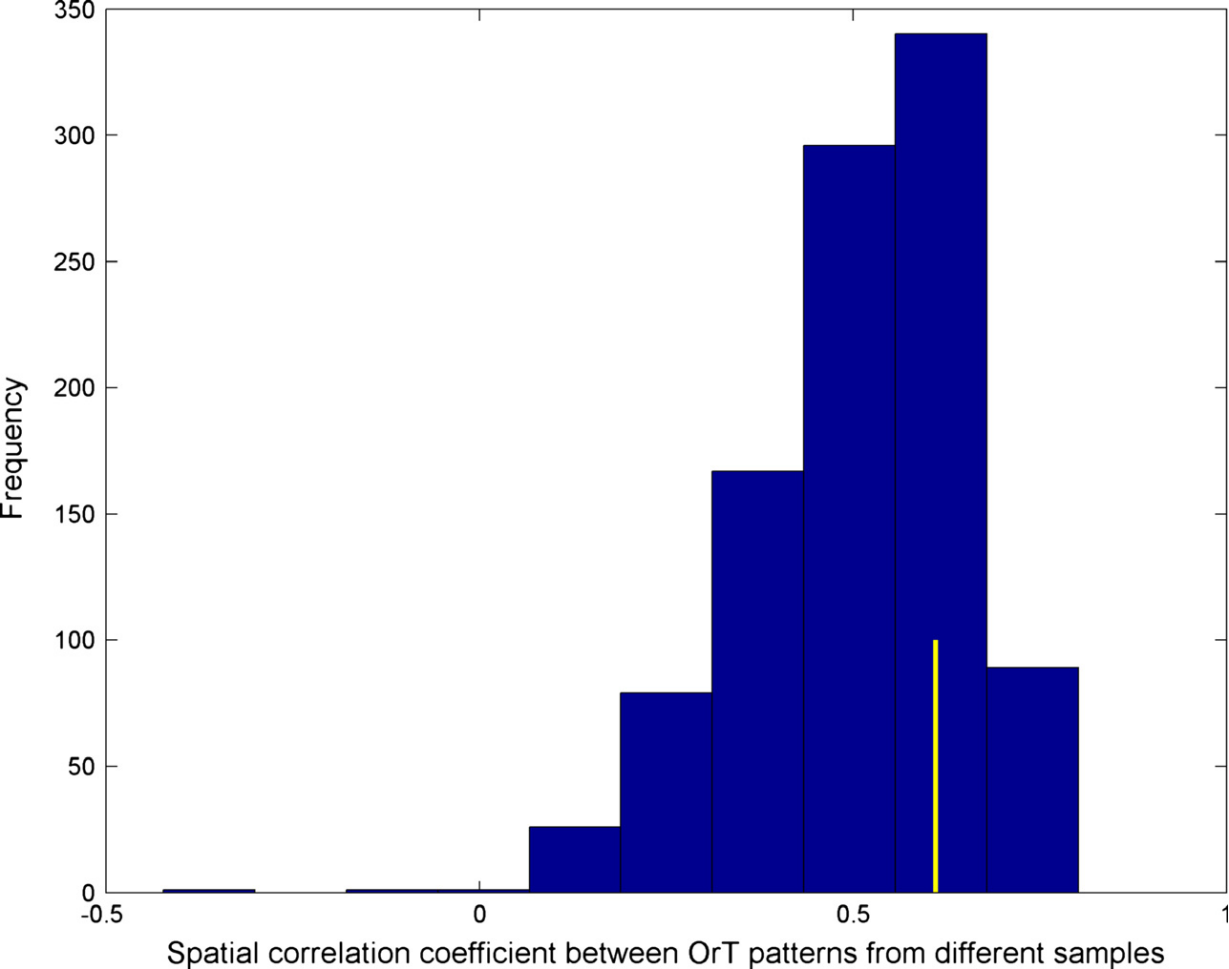
Histogram of Pearson *r* measuring the spatial correlation between Study I and Study II OrT pattern with group membership permuted randomly. The yellow line represents the point estimate (*r* = 0.611) for the spatial correlation between the unpermuted Study I and Study II OrT Patterns. The yellow line being outside of the tail of the histogram demonstrated spatial similarity between the OrT patterns in the two studies.

Tables 1 and 4 show the brain regions most influential in the OrT pattern derived from Study I and II, respectively, using the same intensity threshold and cluster size (*Z* > 1.96, *k* > 50 voxels). Qualitatively comparing the two sets of brain regions, there were overlaps between the two studies, such as BA 9, 18, and 19, but there were also discrepancies such as BA six being in Study II pattern only. To statistically measure the spatial similarity of the two patterns, permutation of group membership between the two studies was performed to generate a null histogram of the spatial correlation values against which the point estimate can be compared. As shown in Figure4, which is the null histogram for Pearson *r* values of the spatial correlation, the point estimate for the spatial correlation is 0.611, which lies in the bin with the highest frequency of values, and thus the spatial similarity between the OrT pattern from the two studies are not significantly dissimilar from each other and the rederivation of OrT pattern in Study II data supported successful replication.

**Table 4:** Brain regions exceeding the threshold of *z* > 1.96 and extent threshold of 50 contiguous voxels in the Study II Ordinal Trend pattern

Region	Lat	BA	*x*	*y*	*z*	*k*
Positive Loading						
Inferior Frontal/Precentral Gyrus	L	9/44	−42	8	24	186
Cerebellum/Fusiform Gyrus	L/R	19	−34	−70	−20	5973
Mid-occipital/Precuneus	L	18/19	−28	−68	42	329
Middle Frontal Gyrus	L	6	−28	14	60	161
Precuneus	L/R	7	2	−64	52	856
Superior Frontal Gyrus	R	8	2	16	54	139
Superior/Middle Frontal Gyrus	R	6/8	30	−4	50	194
Middle Occipital Gyrus	R	19	30	−88	12	98
Negative Loading						
Middle Temporal Gyrus	L	19/39	−42	−64	22	326
Insula	L	13	−38	−18	20	2178
Medial Frontal Gyrus/Ant. Cingulate	L/R	10/32	−8	48	−8	1556
Cingulate Gyrus	L/R	30/31	−6	−48	30	392
Cingulate Gyrus	L	24	−2	−22	36	141
Medial Frontal Gyrus	R	6	4	−28	60	130
Claustrum/Inferior frontal gyrus	R	NA	38	−4	6	3397
Insula/Superior temporal gyrus	R	13	60	−38	20	82

Lat, laterality; BA, Brodmann Area; L, left; R, right; NA, not applicable; *x*,*y*,*z*, MNI coordinates; *k*, cluster size.

## Discussion

This study had two goals: (1) to replicate a previously observed task-related functional pattern from a task-switching paradigm in a new sample of participants, and (2) to specifically examine whether age-related differences in the expression of the functional activation pattern accounts for differences in age-related task performance. Two methods of replication were performed: (1) forward application of the functional pattern, the fMRI OrT pattern identified in Gazes et al. (2012), Study I, to Study II data and the expression scores of the pattern was examined for the likelihood of obtaining by chance the number of participants showing consistently increasing trend from single-task to task-switching condition; and (2) derivation of a new OrT pattern in Study II data for which the statistical significance was gauged by examining the spatial similarity of the patterns between the two studies. Results showed that the OrT pattern was expressed in the new participant group. We further extended the previous result by demonstrating significant mediation of age-related differences in ΔRT through ΔfMRI expression.

### Replication

Successful replication of the relationship of the fMRI pattern expression with task demand and behavioral performance in an independent sample demonstrates the stability of this pattern as a neural substrate underlying the task-switching paradigm. In general, such two-fold replication is by no means guaranteed, even if the relationships discovered in the original data sample are highly statistically significant. The successful replications in the current study hint at two factors: (1) the large magnitude of the induced task-related changes by the task-design and, (2) the power of OrT by focusing on monotonic within-subject changes to extract the true underlying neural correlate of task switching.

Replication was confirmed with two complementary approaches, the first approach tested the replicability of the predictive value of an OrT pattern while the second approach tested the replicability of the spatial pattern derived with OrT CVA. Replication was determined to be successful via both approaches, demonstrating that the original OrT pattern can predict behavior in a new data set and that the OrT can be used to derive spatially comparable patterns in two different data sets. While the OrT pattern from the two studies did not consist of perfectly overlapping brain regions, which is an unrealistic demand on two different sets of data, we showed that the spatial correlation between the two patterns were not statistically different from the set of correlations obtained from randomly permuting group membership, suggesting that the OrT patterns were spatially similar to each other.

The Study I OrT pattern consisted of regions with positive and negative loadings, the sign of which indicates whether activity increases or decreases with increasing task difficulty, respectively. The regions with positive loadings were dominated by areas associated with visual and motor processes such as the cerebellar and bilateral occipital activation while the negatively weighted regions consisted of the prefrontal, parietal, temporal, and striatal cortices, and in the cingulate cortex, regions usually associated with higher cognitive processes. A likely explanation for the apparently counterintuitive directions of activation can be derived from observing the association between the expression scores and RT. Positive association was found between load-related differences in activation pattern expression and RT suggesting that greater load-related increases in activation of the motor and visual areas (positive loadings) and greater decreases in the activity of the higher cognitive regions (negative loadings) during task-switching were correlated with greater increases in RT. When considered in the context of age, positive relationships among age, load-related increases in pattern expression, and RT suggest that older adults showed greater load-related increases in activation for the motor and visual areas, and greater decreases in activation for regions associated with higher cognition than younger adults. The indirect effect of age on ΔRT via delta pattern expression further suggests that older adults' greater reliance on visual and motor processing regions and lowered reliance on higher cognitive processes partially accounted for the age-related differences in ΔRT. Thus, while the OrT pattern may not be the primary task-switching pattern, which explains the counterintuitive directions of activation, it is an age-sensitive pattern that differentiates between the age groups.

As a side note, the ΔRT measure used in our analysis is different from the frequently used measure of global switch cost. Global switch cost is the difference between the mean RT during single-task blocks and during nonswitch trials in switch blocks, which quantifies the additional response time due to retaining two visuomotor response sets in the workspace relative to retaining only one response set active (Kray and Lindenberger 2000). Aging has been found to increase global switch cost in previous task-switching studies (Kray and Lindenberger 2000). The ΔRT used in our analysis quantifies the overall change in response processing rather than a specific task-switching mechanism. ΔRT was used instead of global switch cost as an accurate behavioral counterpart to the fMRI data, which also measured the differences between the entire single-task blocks to the task-switching blocks.

### Neural reserve

Even though the OrT pattern may not be the primary task switching activation pattern, both age groups do express the OrT pattern, with older adults showing higher expression than young adults. The mediation of age-related differences in task performance by differences in brain activation is consistent with the concept of neural reserve which posits that the same brain regions are used from youth to old age but with differing levels of efficiency and capacity (respectively defined as the rate of increase in BOLD activation with increasing task demand, and the maximum activation level or the asymptote of the activation versus task demand curve in Stern 2009). As task difficulty increases within the lower range of task demand, older adults' lower efficiency results in higher fMRI activation relative to young adults, but at higher levels of task demand, which surpasses older adults' processing capacity, the trend reverses with younger adults showing higher activation (Stern et al. 2005; Stern 2009). This pattern has been observed across tasks and fMRI analytical approaches. In Stern et al. (2012), a delayed item recognition task was used with a multivariate covariance analysis of the fMRI data across five task difficulty levels. Sebastian et al. (2013) examined three inhibitory tasks of increasing difficulty with a region of interest approach for the fMRI data analysis. Participants in Toepper et al. (2014) performed spatial working memory task and also employed a region of interest approach for fMRI data analysis. All of these studies reported similar age-related fMRI activation trends as hypothesized by neural reserve.

Similar to the concept of neural reserve, Fabiani's GOLDEN framework (growing of lifelong differences explains normal aging) (2012) postulates that aging processes occur on a continuum such that age-related changes can occur in young age. The mediation result in the current study supports the concept of neural reserve and GOLDEN by showing that an activation pattern was shared by both young and older adults and that differential expression of this pattern accounted for age-related differences in task performance. Variation in the expression of this pattern may be attributed to differences in efficiency as postulated by neural reserve. The high mean accuracy rate indicated that the task was easy for participants, suggesting that processing capacity was not reached and that the difference in pattern expression was due to variability in processing efficiency. While our result does not span the full difficulty levels to capture the reversal of activation levels across the two age groups, the indirect effect of age on ΔRT via ΔfMRI expression demonstrated the usage of the same activation pattern by young and older adults in performing task-switching.

### Visual stimulation coincided with reaction time

Presentation of the activation task stimulus ended upon response, which may have introduced the confound of increased brain activation level with longer reaction time (Grinband et al. 2008) due to greater stimulation in areas of the brain both within and possibly even outside of visual cortices. We performed two empirical checks to examine if longer reaction time was the major influence in the OrT pattern, one checked for spatial influence of the visual cortices and the second checked for overall influence of RT on the OrT pattern.

For the spatial check, we excluded areas of the visual cortices defined in the Automated Anatomical Labeling Atlas (Tzourio-Mazoyer et al. 2002) as Calcarine, Cuneus, Lingual, and Occipital (Superior, Middle, and Inferior) for both hemispheres (12 ROIs) from the OrT pattern then recalculated the pattern expressions for both groups of participants, then tested if there is still a significant Ordinal trend in both data sets. After voxels from the visual cortices were excluded, there were 12 exceptions for Study 1 and 10 exceptions for Study 2, with *P *<* *0.001 in both studies.

**Figure 5:**
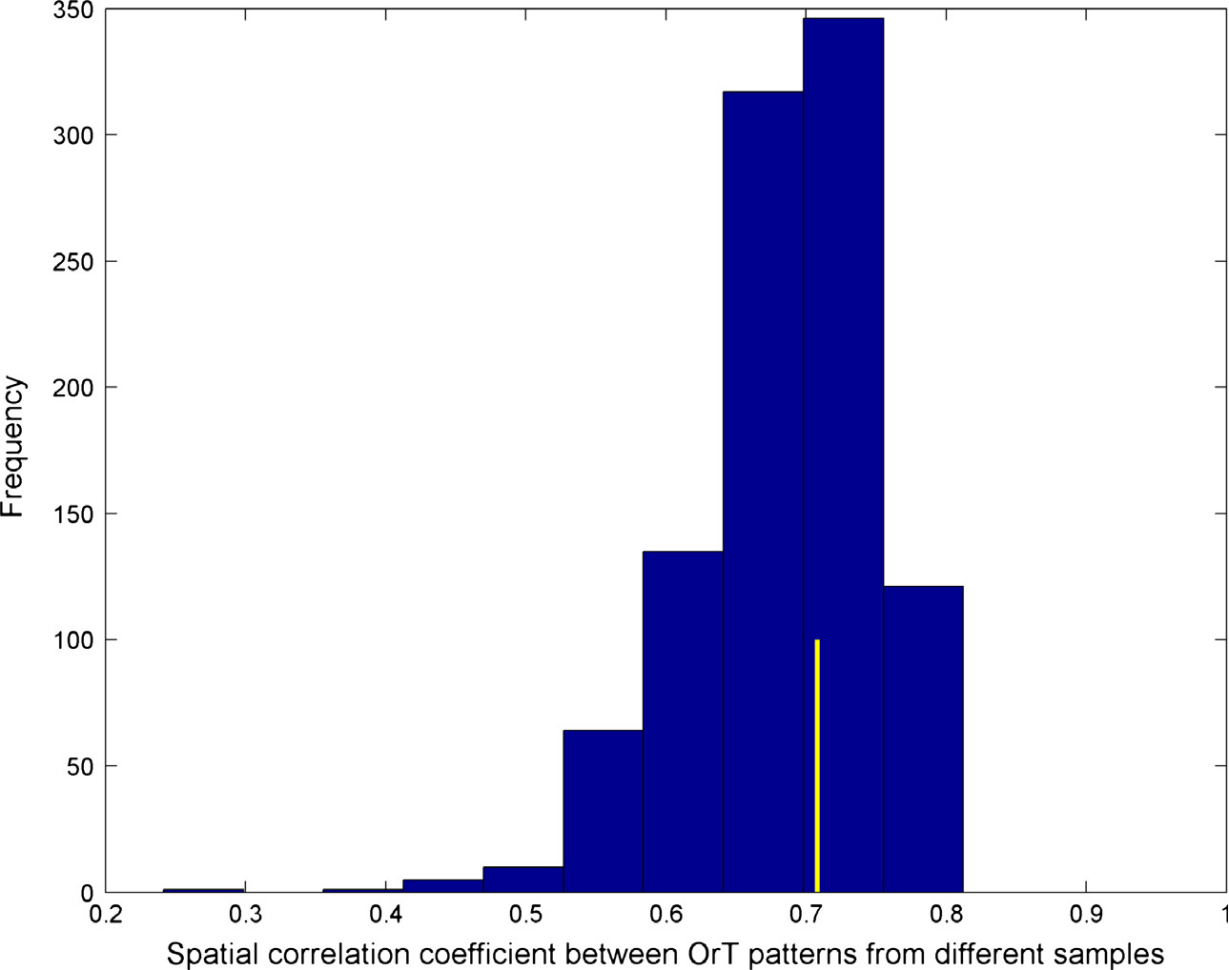
Histogram of Pearson *r* measuring the spatial correlation between the full Study I and the RT-residualized Study II OrT patterns with group membership permuted randomly. The yellow line represents the point estimate (*r* = 0.708). The yellow line being outside of the tail of the histogram demonstrated spatial similarity between the OrT patterns with and without residualizing out RT, thus supporting the minimal influence of RT on the OrT pattern.

Secondly, we partialled out the effect of RT from the fMRI data, then derived an OrT in the RT-residualized data. There were only nine exceptions to the ordinal trend in the resulting OrT pattern (results are not reported here), which is two less than the number of exceptions in the original OrT pattern, demonstrating that even without the influence of RT, brain activation for the majority of participants still show a monotonic increase with greater task difficulty. Furthermore, ΔfMRI expression scores from the RT-residualized OrT pattern also mediated the effect of Age group on ΔRT (indirect effect = 0.0130 with 95% confidence interval [0.0003 0.0334]). We also tested the spatial similarity of the OrT pattern between the full Study 1 patterns with the RT-residualized Study 2 pattern using the permutation approach as described in section 2.6.2. The reason we did not compare the patterns from the same study because comparing Study 1 with Study 2 data would be a more stringent test of spatial similarity. Figure5 shows the histogram of the Pearson *r* values for the permuted data and for the point estimate of *r* = 0.708 (in yellow), which is outside of the tail of the histogram, supporting the similarity of the two patterns. Therefore, it is unlikely that the RT-driven visual stimulation was the primary source of the OrT pattern in our results.
